# Effect of Laparoscopic Sleeve Gastrectomy on Fasting Gastrointestinal, Pancreatic, and Adipose-Derived Hormones and on Non-Esterified Fatty Acids

**DOI:** 10.1007/s11695-016-2302-1

**Published:** 2016-07-27

**Authors:** John E. Farey, Tamara C. Preda, Oliver M. Fisher, Angelique J. Levert-Mignon, Rebecca L. Stewart, Elisabeth Karsten, Benjamin R. Herbert, Michael M. Swarbrick, Reginald V. Lord

**Affiliations:** 1 Department of Surgery, School of Medicine, University of Notre Dame, Sydney, New South Wales Australia; 2 Gastro-oesophageal Cancer Research Program, St Vincent’s Centre for Applied Medical Research, Suite 606, 438 Victoria Street, Darlinghurst, Sydney, NSW 2010 Australia; 3 Diabetes and Metabolism Division, Garvan Institute of Medical Research, Darlinghurst, Sydney, Australia; 4 Biomolecular Frontiers Centre, Department of Chemistry and Biomolecular Science, Faculty of Science, Macquarie University, Sydney, Australia; 5 School of Medical Sciences, UNSW, Sydney, NSW Australia; 6 Centre for Diabetes, Obesity and Endocrinology, The Westmead Institute for Medical Research, The University of Sydney, Westmead, NSW Australia

**Keywords:** Sleeve gastrectomy, Bariatric surgery, C-peptide, Insulin, Glucagon, Ghrelin, GIP, GLP-1, Leptin, NEFA, PAI-1, Resistin, Weight loss

## Abstract

**Background:**

Alterations in gastrointestinal, pancreatic, and adipose hormone levels may have a greater role in weight loss than initially appreciated. The laparoscopic sleeve gastrectomy (LSG) operation is now the most frequently performed bariatric operation in many countries, but there are relatively few data regarding its molecular effects. We sought to characterize the effect of LSG on fasting plasma levels of selected hormones and on non-esterified fatty acids (NEFA), and to compare these to levels in non-obese control individuals.

**Materials and Methods:**

The levels of nine plasma hormones were measured using a multiplex bead-based assay at baseline and at 3 months after operation in 11 obese patients undergoing LSG. NEFA levels were also measured. The levels were compared to those for 22 age- and sex-matched non-obese individuals.

**Results:**

At baseline, obese patients showed significantly higher expression of C-peptide, insulin, and leptin and significantly lower ghrelin, glucose-dependent insulinotropic peptide (GIP), and resistin compared to non-obese controls (*p* < 0.05). LSG resulted in a reduction in BMI from 42.5 ± 6.47 kg/m^2^ at operation to 35.2 ± 5.14 kg/m^2^ at 3 months (42 % mean excess weight loss, *p* < 0.001). LSG led to a significant decrease in ghrelin, glucagon-like peptide-1 (GLP-1), glucagon, leptin, plasminogen activator inhibitor-1 (PAI-1), and NEFA.

**Conclusion:**

LSG induces marked early changes in the fasting levels of factors thought to be important regulators of obesity and metabolic health. These changes differ somewhat from the findings for operations with a malabsorptive component, suggesting that subtle differences exist in the mechanisms of weight loss between LSG and other bariatric operations.

## Introduction

Bariatric surgery is an appropriate, effective treatment for patients with severe obesity and related comorbidities who have been unable to gain adequate benefits from conservative measures such as diet and exercise [1–3]. While initially thought to function by simply limiting energy intake [4], it is now clear that both the restrictive and malabsorptive components of bariatric operations induce weight loss by altering the hormonal milieu of the gut and visceral adipose tissue, among other factors such as increased circulating bile acids [5, 6]. Most notably, the expression of several hormones relating to the regulation of appetite and energy expenditure have been observed to have changed following surgical weight loss, of which most attention has been devoted to ghrelin, leptin, adiponectin, glucagon-like peptide 1 (GLP-1), peptide YY, and glucagon. Understanding the mechanism of action of the various bariatric surgical techniques is crucial for gaining a clearer picture of the pathophysiology of obesity and may allow for the development of less invasive methods of achieving weight loss.

The laparoscopic sleeve gastrectomy (LSG) operation is gaining acceptance as the bariatric procedure of choice for many patients and surgeons due to its simplicity, safety profile, and competitive efficacy compared to more complex operations such as the Roux-en-Y gastric bypass (RYGB) [7–9]. It has been hypothesized that LSG results in acute alterations to the regulation of appetite and mechanical function of the gut, both directly via excision of the greater curvature of the stomach, and indirectly through downstream endocrine and nutrient processing changes, all of which act to promote prolonged weight reduction [5]. Prior studies have demonstrated decreased levels of leptin and ghrelin [10, 11] and increased GLP-1 [12] following LSG, but much less is known about other molecular factors implicated in the regulation of blood-glucose homeostasis, gastric function, and emerging markers of adiposity. Additionally, many studies have sought to measure these hormones as surrogate markers of efficacy to evaluate LSG compared to other bariatric techniques [13], rather than to ascertain the mechanism of action of LSG.

This study was designed to characterize the changes in a series of gastrointestinal, pancreatic, and adipose hormones as well as non-esterified fatty acids (NEFA) before and after LSG in obese patients and additionally to compare these to baseline levels in non-obese control individuals. These findings contribute to the knowledge base regarding the biological effects of the LSG operation.

## Methods

### Study Design and Study Population

We performed a prospective study consisting of obese patients undergoing elective LSG with a single surgeon (RVL) with non-obese individuals as controls. Inclusion criteria for obese participants were prolonged failure of conservative therapy, age > 18 years, and BMI > 35 kg/m^2^ with obesity-related comorbidities or BMI > 40 kg/m^2^. Inclusion criteria for non-obese controls were BMI < 30 kg/m^2^. Non-obese controls were age- and sex-matched in a 2:1 (control to patient) ratio and selected from ambulatory clinics where they presented for elective endoscopy. Exclusion criteria for non-obese controls were any past history of bariatric surgery, current adherence to a calorie-restricted diet or use of therapeutic weight loss medication, and any history of malignancy. Caloric intake was not calculated.

Obese participants were scheduled for two study visits. At each study visit, anthropometric data, including height, weight, BMI, and excess body weight (EBW), were measured with patients barefoot. BMI was calculated as weight/height × height (kg/m^2^). EBW was calculated as weight above BMI of 25 kg/m^2^ and converted to kilograms. Percentage excess body weight loss (%EBWL) was calculated as (EBW_baseline_ − EBW_follow-up_/EBW_baseline_) × 100. Patients were placed on an isocaloric diet for at least 2 weeks prior to LSG. This diet was ceased before the blood collection at 3 months post-operatively. Clinical data, including comorbidities and medication use, were extracted from patients’ medical records. Blood samples from non-obese controls were acquired once during their scheduled visit, prior to endoscopy or administration of sedative agents. Institutional review board approval was obtained for this study, and all patients provided written informed consent.

### Blood Collection and Preparation

Blood was collected from fasting participants into two 10-mL EDTA tubes for blood glucose and the gut hormone measurements at study visits. Both the participants and control individuals had been fasting for at least 6 h prior to all study blood collection. Immediately after collection, each tube was gently mixed by inverting 10 times. Tubes were spun in a benchtop centrifuge at 3000 rpm (1400×*g*) for 10 min, and the plasma was transferred into cryotubes and stored at −80 °C until analysis.

### Surgical Procedure

In brief, the LSG operation was performed using five laparoscopic port sites with induction of a pneumoperitoneum. A hiatus hernia, if present, was repaired using interrupted posterior crural sutures (0 Ethibond (Ethicon, Cincinnati OH), with or without felt pledgets). The Ligasure blunt tip device (Medtronic, Minneapolis MN, USA) was used to divide vessels along the greater curvature. The Echelon linear cutting device (Ethicon, Cincinnati OH) was used to divide the stomach along a line parallel to the lesser curvature from a point 4–6 cm proximal to the pylorus on the greater curvature to a point 1–2 cm lateral to the gastroesophageal junction. The LSG operation was performed over 32-Fr bougies for females and 36-Fr bougies for males. There was no operative mortality and no morbidity.

Gut hormones were analyzed using a magnetic bead-based assay (BioRad, Hercules, CA, USA). The Bio-Plex Pro Human Diabetes Assay (171A7001M) detected fasting levels of C-peptide, ghrelin, glucose-dependent insulinotropic peptide (GIP), glucagon-like peptide-1 (GLP-1), glucagon, insulin, leptin, plasminogen activator inhibitor-1 (PAI-1), and resistin (also known as adipose tissue-specific secretory factor (ADSF)). Fasting gut hormone levels were quantified at the Australian Proteome Analysis Facility at Macquarie University, Sydney, Australia, according to an optimized process described previously [14]. Samples were run in duplicate on a 96-well plate using 50 μL of neat plasma using the Bio-Plex 200 instrument (BioRad, Hercules, CA, USA).

Non-esterified fatty acids (NEFA) were measured in freshly thawed plasma using a commercially available assay (HR Series NEFA-HR [2]) according to the manufacturer’s instructions (Wako Diagnostics, Richmond, VA, USA). In brief, a standard curve from 0.3125 to 10 nmol (in 5 uL volume) was made by serial dilution in a 96-well plate followed by adding 5 uL of each vortexed plasma sample, addition of the manufacturer’s reagents, and measuring absorbance at 550 nm in a plate reader.

### Statistical Analysis

Continuous variables were compared using student’s *t* test, Wilcoxon rank-sum test, one-way analysis of variance, or the Kruskal–Wallis test and performed as paired or unpaired analyses where appropriate. Differences between proportions derived from categorical data were analyzed using Pearson’s chi-squared test or Fisher’s exact test where appropriate. Data are presented as median with interquartile range (IQR) unless denoted otherwise. All *p* values <0.05 were considered statistically significant. All analyses were performed using R version 3.2.2. (R Foundation for Statistical Computing, Vienna, Austria), and Prism (GraphPad Prism version 6.0c for Mac OS X, GraphPad Software, San Diego, CA, USA) was used for graphs.

## Results

### Participant Characteristics

Twenty-two non-obese control individuals undergoing elective outpatient upper gastrointestinal endoscopy for the investigation of gastroesophageal reflux symptoms (19 patients) or surveillance of Barrett’s esophagus (3 patients) and 11 obese patients scheduled to undergo LSG were prospectively recruited. The composition of the groups was not significantly different for gender and age, providing a 2:1 age- and sex-matched control to patient ratio (Table 1). Metabolic studies were performed following a 10-h overnight fast at baseline and at 3 months after LSG. All participants receiving medications for type 2 diabetes mellitus (T2DM) withheld them on the morning of the blood test. Anthropometric, clinical, metabolic, and obesity-related comorbidity variables at baseline and 3 months after surgery are shown in Table 1. As expected, obese participants had significantly higher BMI and body weight than non-obese controls, both at baseline and at follow-up.

**Table 1:** Demographic, anthropometric, and obesity-related comorbid illness variables in obese participants at baseline and 12 weeks after laparoscopic sleeve gastrectomy

Variable	Non-obese controls	Obese group baseline	Obese group 12 weeks after LSG*	*p* value**
Demographics				
Male	12	6	6	–
Female	10	5	5	–
Age (years)	50.45 ± 10.67	51.55 ± 12.81	–	–
Clinical				
Weight (kg)	67.43 ± 12.81	119.70 ± 27.76	99.84 ± 21.27	<0.001
BMI (kg/m^2^)	22.72 ± 2.58	42.48 ± 6.47	35.18 ± 5.14	<0.001
EBW (kg)	–	50.09 ± 20.66	29.08 ± 16.19	<0.001
Weight loss (kg)	–	–	19.86 ± 8.09	–
EBWL (%)	–	–	42 ± 9	–
Metabolic				
Fasting glucose (mmol/L)	NA	6.07 ± 2.26	5.15 ± 1.10	0.28
Obesity-related comorbidities				
Type-2 diabetes mellitus		5	2	0.28
Hypertension		5	3	0.41
Hypercholesterolemia		2	2	–
Obstructive sleep apnea		9	7	0.08
Polycystic ovarian syndrome		1	1	–
Gastroesophageal reflux disease		3	1	0.05

Data are reported as mean ± SD

*LSG laparoscopic sleeve gastrectomy

**p values are for pre- vs. post-operative comparison of obese patients

According to patient history and an examination of correspondence from their referring primary care physicians, some of the control group individuals had cardiovascular risk factors, specifically, four patients had a history of hypertension, two patients had T2DM treated by oral antihyperglycemic medications (1 patient) or insulin (1 patient), and two patients had dyslipidemia. Five of the obese participants had a history of T2DM. Medications were documented at each visit. Metformin was continued post-operatively regardless of post-operative glucose levels.

### Weight Loss Following LSG

There were significant reductions in weight, BMI, and fasting glucose following LSG (Table 1). At the median follow-up interval of 12 weeks, mean BMI reduction was 7.3 kg/m^2^ (SD 2.5 kg/m^2^, Table 1).

### Fasting Hormone Levels in Pre-LSG Obese Patients Compared to Non-Obese Control Individuals

**Fig. 1:**
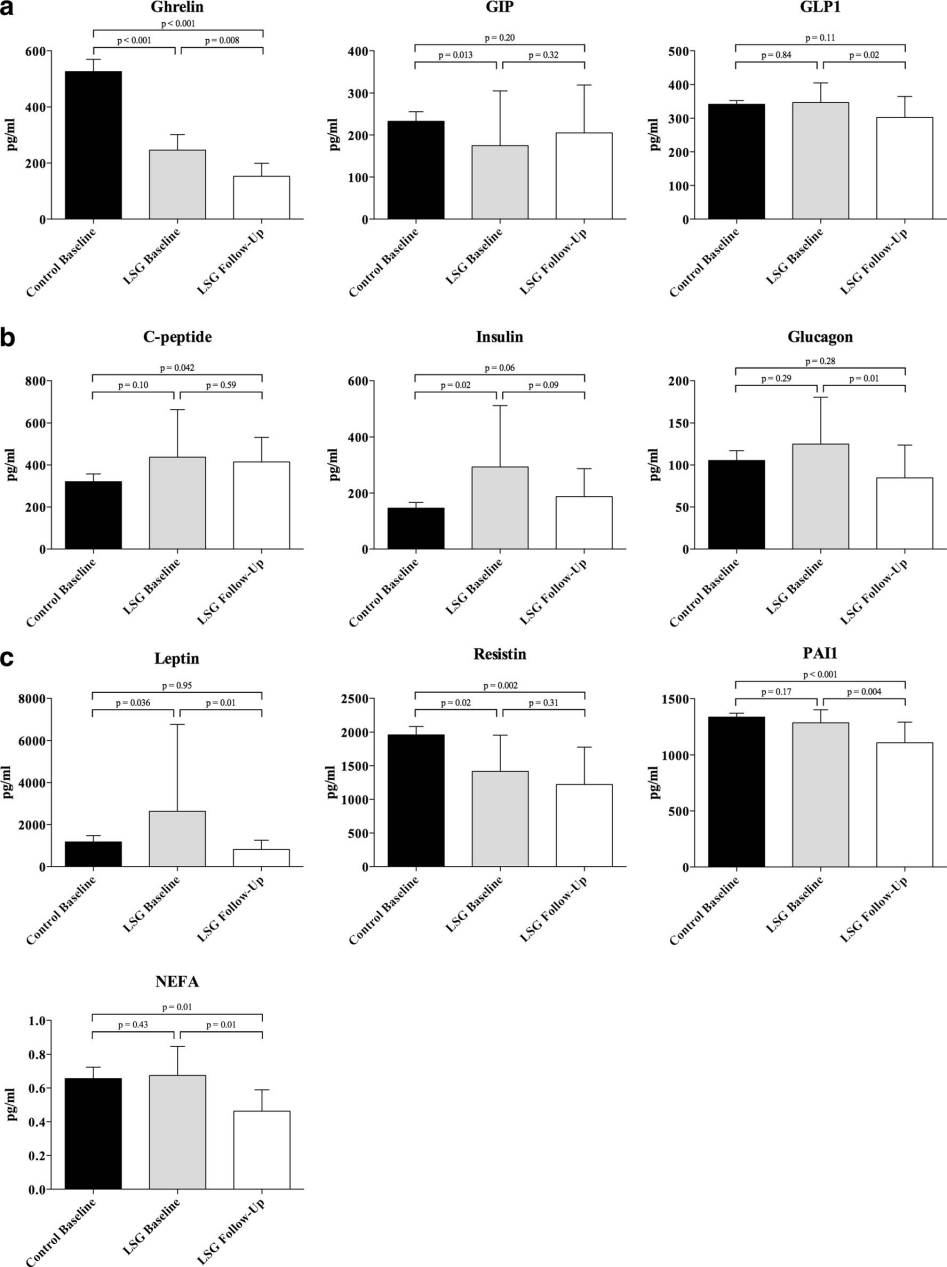
Comparison of fasting levels of 10 fasting factors between obese participants and healthy weight controls, both at baseline and 12 weeks follow-up following laparoscopic sleeve gastrectomy (*LSG*). **a** Gastrointestinal hormones ghrelin, gastric inhibitor peptide (*GIP*), and glucagon-like peptide-1 (*GLP-1*). **b** Pancreatic endocrine products C-peptide, insulin, and glucagon. **c** Adipose tissue hormones leptin, plasminogen activator inhibitor-1 (*PAI-1*), resistin, and non-esterified fatty acids (*NEFA*)

Baseline fasting plasma levels of the 10 factors in obese patients and non-obese control individuals are shown in Table 2 and Fig. 1. As shown, obese patients had significantly higher fasting levels of insulin and leptin and significantly lower levels of ghrelin, GIP, and resistin, compared to non-obese controls. There were no significant differences between the groups for the other factors.

**Table 2:** Fasting levels of gastrointestinal, pancreatic, and adipose-derived hormones as well as non-esterified fatty acids in healthy weight controls and obese participants at baseline

Variable	Non-obese group baseline	Obese group baseline	*p* value*
C-peptide	304.6 (234.2–417.5)	442.6 (261.1–475.3)	0.10
Ghrelin	470.4 (380.0–632.5)	240.3 (212.4–310.1)	<0.001
GIP	211.8 (177.9–236.3)	140.2 (88.24–205.2)	0.013
GLP-1	332.7 (311.0–367.5)	338.9 (314.6–363.5)	0.84
Glucagon	89.86 (69.58–145.4)	106.4 (82.63–179.9)	0.29
Insulin	113.7 (90.38–171.8)	239.8 (132.1–383.6)	0.024
NEFA	0.60 (0.51–0.77)	0.71 (0.49–0.82)	0.43
Leptin	687.7 (476.7–1308.0)	1251.0 (1096.0–2312.0)	0.036
PAI-1	1390.0 (1243.0–1437.0)	1311.0 (1188.0–1323.0)	0.17
Resistin	2010.0 (1589.0–2442.0)	1230 (1018.0–1831.0)	0.02

Data are reported as median (IQR [25th, 75th percentile]). All values are pg/mL except for NEFA, which is shown as mmol/L

*As determined through unpaired Mann-Whitney *U* test

### Changes in Fasting Factor Levels Following LSG

Fasting levels of the 10 factors were repeated 3 months after LSG to identify changes associated with weight loss (Table 3 and Fig. 1). At follow-up, the fasting levels of ghrelin, GLP-1, glucagon, leptin, PAI-1, and NEFA were all significantly decreased compared to preoperative baseline levels (Table 3). There were non-significant decreases in C-peptide, insulin, and resistin and a non-significant increase in GIP.

**Table 3:** Fasting levels of gastrointestinal, pancreatic, and adipose-derived hormones as well as non-esterified fatty acids in obese participants at baseline and 12 weeks after laparoscopic sleeve gastrectomy

Variable	Obese group baseline	Obese group 12 weeks after surgery	*p* value*
C-peptide	442.6 (261.1–475.3)	378.4 (314.2–483.4)	0.59
Ghrelin	240.3 (212.4–310.1)	135.4 (111.8–193.2)	0.008
GIP	140.2 (88.24–205.2)	152.5 (104.3–319.4)	0.32
GLP-1	338.9 (314.6–363.5)	269.7 (255.4–352.1)	0.02
Glucagon	106.4 (82.63–179.9)	69.69 (58.80–118.0)	0.01
Insulin	239.8 (132.1–383.6)	155.5 (117.5–296.3)	0.09
NEFA	0.71 (0.49–0.82)	0.51 (0.38–0.54)	0.01
Leptin	1251.0 (1096.0–2312.0)	637.4 (500.3–1149.0)	0.01
PAI-1	1311.0 (1188.0–1323.0)	1130.0 (1090–1264)	0.004
Resistin	1230 (1018.0–1831.0)	978.2 (749.8–1724.0)	0.31

Data are reported as median (IQR [25th, 75th percentile]). All values are pg/mL except for NEFA, which is shown as mmol/L

*As determined through paired Mann-Whitney *U* test

### Fasting Factor Levels in Patients Following LSG Compared to Non-Obese Control Individuals

After LSG induced weight loss, patients had significantly lower levels of ghrelin (*p* < 0.001), NEFA (*p* = 0.01), PAI-1 (*p* < 0.001), and resistin (*p* = 0.002), compared to non-obese control individuals. Of these, ghrelin and resistin were significantly lower pre-LSG (Table 2) but decreased further after LSG, and NEFA and PAI-1 had not been significantly different in pre-LSG patients compared to non-obese controls. Of the factors that were higher pre-LSG compared to non-obese control individuals (insulin, C-peptide, leptin; Table 2), C-peptide (*p* = 0.042) became significantly higher whereas insulin (*p* = 0.06) and leptin (*p* = 0.95) levels returned to similar ranges to non-obese control levels and glucagon remained not significantly different (*p* = 0.28).

Figure 1 shows the gastrointestinal hormones (Fig. 1a), pancreatic hormones (Fig. 1b), and adipose tissue-derived hormones and non-esterified fatty acid results (Fig. 1c) for the non-obese control individuals and at baseline pre-LSG and at 12 weeks following LSG for the obese patients.

## Discussion

In this study, we observed the differences between obese and non-obese participants in fasting levels of some gastrointestinal, pancreatic, and adipose tissue hormones and studied the changes that occurred with successful weight loss following LSG. We showed that LSG results in significant reductions in most of these factors: two gastrointestinal hormones (ghrelin and glucagon-like peptide-1 [GLP-1]), two pancreatic hormones (insulin and glucagon), two adipose tissue-derived hormones or adipokines (leptin and plasminogen activator inhibitor-1 [PAI-1]), and non-esterified fatty acids (NEFA).

A remarkable finding of this study was that at only 12 weeks after LSG, the plasma levels of all the six factors that had significantly decreased following surgery were lower than the levels present in non-obese control individuals. This was the case despite the post-LSG patients still being severely obese at 12 weeks, prompting the interpretation that the hormonal changes resulting from surgery, and also potentially from caloric restriction, may partly mediate the weight loss and associated metabolic improvements [15]. This study may thus provide insights into the molecular mechanisms that contribute to the beneficial effects of LSG and how these mechanisms differ from those reported for other bariatric operations.

Of the gastrointestinal tract hormones studied, the reduction in plasma levels of ghrelin is expected after excision of the greater curvature and gastric fundus of the stomach. Produced by the X/A-like cells during fasting [16], ghrelin stimulates food intake via neuropeptide Y-AgRP neurons in the arcuate nucleus, vagus nerve, and brainstem and the reduced appetite after LSG is often attributed to lower ghrelin levels. Our findings of both lower ghrelin levels in obese patients compared to healthy weight volunteers and a decrease in ghrelin levels with weight loss following LSG, are in accordance with previous studies [10, 13, 17–19]. Doubt has emerged, however, as to whether ghrelin is truly an important mediator of weight loss; LSG was equally effective in ghrelin deficient mice, suggesting that weight loss may be independent of decreased serum levels of the hormone [20].

We found a reduction in levels of glucagon-like peptide-1 (GLP-1), a hormone produced in the intestinal epithelial L-cells in response to a meal via differential processing of the proglucagon gene [21], after LSG. GLP-1’s function as an incretin, acting to stimulate insulin secretion and inhibit glucagon secretion, has been exploited pharmacologically for the purposes of limiting post-prandial hyperglycemia in T2DM [22], and GLP-1 receptor agonists have been demonstrated to promote weight loss [23]. Additionally, a role in the physiological regulation of appetite and food intake has been proposed [24].

Most commonly, an exaggerated GLP-1 secretion in response to a meal has been observed following LSG [10, 13, 17] and has been suggested as contributing to weight loss by promoting early satiety and reduced food intake. We did not assess post-prandial GLP-1 response in this study; however, our results suggest that the improved insulin sensitivity and blood-glucose homeostasis may result in the downregulation of the products of the proglucagon gene, such as GLP-1. Whether this finding is significant in terms of LSG inducing weight loss is unclear, LSG has been shown to be equally effective in GLP-1 receptor knockout mice [25] and similar to Dimitriadis et al. [10] we demonstrated no difference in fasting levels of GLP-1 between obese and non-obese controls at baseline. Whether an active form of GLP-1 or a metabolite is being measured may also influence study results and limit comparison between studies [26]. It is therefore difficult to provide firm conclusions at this stage regarding LSG and GLP-1.

The third gastrointestinal hormone studied, glucose-dependent insulinotropic peptide (GIP), is an incretin released from the K cells of the small intestine in response to glucose or fat ingestion to potentiate the glucose-dependent insulin response. GIP contributes to obesity by promoting energy storage through various anabolic effects on adipose tissue [26]; GIP receptor inhibition reduces adipocyte mass and prevents obesity [27, 28]. GIP is released in tandem with GLP-1 and it is thus somewhat unexpected that LSG had a significant lowering effect on GLP-1 but not GIP plasma levels. As in our study, Romero et al. [29] found no change in fasting GIP plasma values after LSG (or RYGB), but the GIP response to a standard mixed liquid meal was significantly increased after LSG (but not RYGB). Surprisingly, the GIP response post-surgery was not different to that of matched obese diabetic non-surgery control individuals in Romero et al.’s study [29]. GIP may be more important for bariatric operations with a malabsorptive component, as supported by our results and by reports that GIP expression is reliably decreased after biliopancreatic diversion, occasionally after RYGB, but not after laparoscopic adjustable gastric banding [30].

An enduring reversal of insulin resistance has been reported for more than half of patients who undergo LSG [31, 32]. We studied three pancreatic hormones (insulin, glucagon, C-peptide) related to insulin resistance. In a study with many more T2DM patients undergoing RYGB, a highly significant fall in C-peptide was observed and pre-surgical levels helped predict the likelihood of diabetes remission [33]. Only five of the 11 LSG patients in our study had T2DM, which may explain our finding of a non-significant change for insulin and for C-peptide we observed, levels of which are generally proportionally secreted [34].

Consistent with improved glycemic control after LSG, we found that surgery resulted in significantly reduced levels of glucagon, a peptide hormone released by pancreatic alpha cells that stimulate glucose release. Similar findings have been reported for RYGB [35, 36]. Another study demonstrated an early attenuation of the glucagon response to a mixed meal early after both LSG and RYGB, but fasting glucagon levels were similar to preoperative values at 1 year after either operation type [13]. Rodent models of LSG also find reduced fasting glucagon, as well as an augmented response following meal stimulation and improved glucagon: insulin ratio that is superior to RYGB [37]. Considered together with our results, most data suggest that LSG provides early and ongoing significant improvement in glucagon levels in both overt clinical T2DM and subclinical insulin resistance in the obese.

Adipose tissue-derived hormones, or adipokines, have been proposed to promote low-grade inflammation in the obese, as well as a dysregulated metabolic state that favors long-term weight regain after intensive medical therapy [38–41]. Our results for leptin and PAI-1 are similar to other reports that LSG leads to significant reductions of both markers of obesity [12, 18]. Animal and human studies have linked production of the adipokine resistin to insulin resistance in the obese [42], and increased expression has been correlated with increasing central obesity [43]. Decreased resistin expression has been demonstrated for RYGB at an identical time period [44]; however, our findings failed to replicate this change for LSG. This suggests that downregulation of the resistin gene may not be an important mediator of weight loss in LSG and that the two procedures differ in their mechanisms of action.

We found that LSG leads to a significant reduction of NEFA. NEFA are formed from the hydrolysis of triglyceride molecules within adipocytes and released into the circulation during negative energy balance [36], consistent with previous studies showing an increase in NEFA concentrations at 1 month post-RYGB [45, 46]. NEFA levels are decreased at 12 and 15 months after RYGB [45, 46], and our study shows that this decrease has occurred by 3 months after LSG. Elevated NEFA concentrations have been demonstrated to downregulate the expression of several lipolysis-promoting enzymes, such as hormone-sensitive lipase and adipose triglyceride lipase [47, 48], coded for by the LIPE and PNPLA2 genes respectively. The magnitude of the change we observed was greater than that observed after RYGB, suggesting that upregulation of the above genes to promote lipolysis is an important mediator of weight loss following LSG. Traditionally, elevated NEFA concentrations in the obese were thought to arise from increased adipose tissue mass, but this assertion has been dismissed in recent years; a systematic review found no difference in fasting levels between the obese and non-obese, consistent with our findings [49].

Limitations of the present study include the relatively small sample size, incomplete characterization of the control group, lack of detail on caloric count and physical activity, and heterogeneity of the obese patient group. A longer follow-up time would more reliably corroborate the changes we observed. Nevertheless, our findings are consistent with the changes in levels of these hormones observed in similar studies, including those at up to 12 months of follow-up. Of our non-obese controls, six have a BMI in the range of 25–26 kg/m^2^ reflecting the availability of controls during the study period and are thus classed as mildly overweight [50]; the mean BMI was, however, well within the healthy range at 22.72 kg/m^2^. Additionally, we did not assess post-prandial secretion of the incretin hormones (GIP and GLP1) in response to a standardized meal test. To do so would have allowed us to investigate dynamic changes to nutrient metabolism that have been previously documented for the LSG procedure [2]. It is known that pre- and post-prandial secretion of incretin hormones in diabetic patients are altered compared to normal controls [3, 4], making it difficult to interpret the true significance of decreased fasting GLP-1 in a heterogeneous obese patient group (5/11 patients had T2DM).

In summary, this study provides further support for the importance of the molecular effects of bariatric operations. We found that at only 3 months after LSG, despite patients still being on average severely obese, most of the factors studied had lowered significantly and were even lower than those found in non-obese individuals. These changes may help explain the rapid improvement in insulin resistance observed after LSG, which occurs for many patients before any significant weight loss, as well as the very early reduction in appetite that is regularly observed. In the absence of a malabsorptive operation study arm, we are unable to make any firm conclusions, but our results suggest that mechanism of action of LSG may differ from operations such as RYGB. Elucidating the molecular benefits of LSG and other bariatric operations offers the prospect of bariatric surgery that is tailored to each individual as well as the hoped-for development of less invasive treatment options.
